# A Printable Magnetic-Responsive Iron Oxide Nanoparticle (ION)-Gelatin Methacryloyl (GelMA) Ink for Soft Bioactuator/Robot Applications

**DOI:** 10.3390/polym16010025

**Published:** 2023-12-20

**Authors:** Han-Wen Yang, Nien-Tzu Yeh, Tzu-Ching Chen, Yu-Chun Yeh, I-Chi Lee, Yi-Chen Ethan Li

**Affiliations:** 1 Department of Chemical Engineering, Feng Chia University, Taichung 40724, Taiwan; hw89965@gmail.com (H.-W.Y.); aliceyipyang@gmail.com (N.-T.Y.); celia403cc@gmail.com (T.-C.C.); 2 Department of Biomedical Engineering and Environmental Sciences, National Tsing Hua University, Hsinchu 300044, Taiwan; zxcvb890717@gmail.com

**Keywords:** magnetic-responsive materials, soft bioactuators/robots, DIW printing, gelatin methacryloyl (GelMA), iron oxide nanoparticles (IONs)

## Abstract

The features or actuation behaviors of nature’s creatures provide concepts for the development of biomimetic soft bioactuators/robots with stimuli-responsive capabilities, design convenience, and environmental adaptivity in various fields. *Mimosa pudica* is a mechanically responsive plant that can convert pressure to the motion of leaves. When the leaves receive pressure, the occurrence of asymmetric turgor in the extensor and flexor sides of the pulvinus from redistributing the water in the pulvinus causes the bending of the pulvinus. Inspired by the actuation of *Mimosa pudica*, designing soft bioactuators can convert external stimulations to driving forces for the actuation of constructs which has been receiving increased attention and has potential applications in many fields. 4D printing technology has emerged as a new strategy for creating versatile soft bioactuators/robots by integrating printing technologies with stimuli-responsive materials. In this study, we developed a hybrid ink by combining gelatin methacryloyl (GelMA) polymers with iron oxide nanoparticles (IONs). This hybrid ION-GelMA ink exhibits tunable rheology, controllable mechanical properties, magnetic-responsive behaviors, and printability by integrating the internal metal ion-polymeric chain interactions and photo-crosslinking chemistries. This design offers the inks a dual crosslink mechanism combining the advantages of photocrosslinking and ionic crosslinking to rapidly form the construct within 60 s of UV exposure time. In addition, the magnetic-responsive actuation of ION-GelMA constructs can be regulated by different ION concentrations (0–10%). Furthermore, we used the ION-GelMA inks to fabricate a *Mimosa pudica*-like soft bioactuator through a mold casting method and a direct-ink-writing (DIW) printing technology. Obviously, the pinnule leaf structure of printed constructs presents a continuous reversible shape transformation in an air phase without any liquid as a medium, which can mimic the motion characteristics of natural creatures. At the same time, compared to the model casting process, the DIW printed bioactuators show a more refined and biomimetic transformation shape that closely resembles the movement of the pinnule leaf of *Mimosa pudica* in response to stimulation. Overall, this study indicates the proof of concept and the potential prospect of magnetic-responsive ION-GelMA inks for the rapid prototyping of biomimetic soft bioactuators/robots with untethered non-contact magneto-actuations.

## 1. Introduction

Actuators are widely applied in various applications, such as clean energy, construction, industrial machinery, aerospace, etc., because actuators can drive objects to perform versatile predetermined actions by converting kinetic energy into motion. In general, actuators can be divided into electric-driven actuators, pressure-driven actuators, magnetic-driven actuators, and others according to the source of their driving force [1]. Currently, soft actuators have been receiving increased attention and have potential applications in the micromanipulation and tissue engineering fields [2]. Compared with conventional actuators made from hard materials, flexible elastomers, and polymeric materials are usually used to fabricate soft actuators and endow them with environmental compliance, deformability, and lightweight properties [3]. These properties allowed soft actuators to be developed and applied in soft robots, wearable devices, or soft grippers fields [4]. Furthermore, according to the trend in the rapid development and recent advances in actuators for biomedical applications, their designs are moving toward small volumes and high degrees of freedom in motion for operating in confined spaces [5]. Actuators that involve biomedical devices, called bioactuators, are promising for bridging the gap between some in vitro and in vivo bio-related uses [6]. These bio-related benefits include artificial muscle for reconstructing/recovering tissues from a sports injury [7,8], drug carriers for controlled release in disease treatment [9,10], biosensors for the real-time monitoring of biochemical or physiological changes in the human body [11,12], and other applications [13,14]. Therefore, the combination of suitable physiochemical properties of materials and well-designed structures provides a novel strategy to trigger the development of new materials or technologies to design bio-oriented actuators or robots with reliable functions in emerging biomedical applications.

In terms of bioactuators, many designs are inspired by biological features, such as tissue movement in the human body and the biological structures of animals, reptiles, insects, or plants. Noticeably, the biological features of natural creatures are usually composed of small repeating units with well-defined complex architectures. However, conventional manufacturing technologies, such as molding or lithography techniques, require complex processes to replicate small and repeating complex natural architectures [15]. Recently, additive manufacturing technology, also called 3D printing technology, has provided a rapid, user-friendly, and stable strategy to fabricate complex architectures through a layer-by-layer stacking procedure. Therefore, 3D printing technologies have been widely used for making different bioactuators with complex structures [15]. For example, Sadeghi and co-workers integrated elastomeric materials and soft sensors into a plant-inspired tropic bioactuator with printed tubular structures which can mimic the growing movement of a plant root [16]. This design can create an isolated channel to provide a possible bending structure for the transmission of food, medicine, or oxygen in rescue scenarios [16]. Truby et al. used silicone elastomers to 3D print a soft robotic gripper for mimicking human hand skills by connecting with a somatosensory system, which could grab and hold a ball by collecting somatosensory feedback [17]. Furthermore, Michael and co-workers 3D printed a soft octopus robot containing a microfluidic system, a catalytic reaction chamber, fuel reservoirs, and pneumatic actuators [18]. The printed soft octopus robot can move driven by a microfluidic logic circuit and the gas generated from a catalytic reaction in the fuel reservoirs. These results indicate the potential applications of 3D printing techniques in fabricating soft actuators or robots. In the past five years, combining 3D printing technology with exogenous stimulation has been used to develop a new printing technique, 4D printing, to achieve the untethered operation of soft bioactuators or robots. Generally, the sources of exogenous stimulation for 4D printing techniques are divided into the changes in external electricity, magnetic forces, light, temperature, humidity, and pH values in the environment. These stimulations endow the printed objects with versatile transformation abilities to achieve the desired functions for different applications [19]. For example, Cao and co-workers developed a thermoplastic rubber-carbonyl iron particle hybrid ink to create magnetic-responsive biomimetic actuators with butterfly and flower-like structures through a 3D fused deposition modeling printing process [20]. The results show that the printed butterfly like structure can flap its wings and the flower-like structure shows a dynamic opening-closing movement of petals under different magnetic field strengths. In addition, Shin et al. engineered a living soft robot inspired by a batoid fish [21]. In the study, they used a carbon nanotube-based polymeric ink to directly print a batoid fish-like structure on an Au electrode-laden polymer layer. Afterward, cardiomyocytes were seeded on the printed batoid structure to obtain a cell-laden biorobot. Furthermore, the soft biorobot shows a swimmer-like movement when treated with an appropriate voltage and frequencies. These reports indicate biosensors combined with stimuli-responsive functions that can expand the applications of bioactuators or soft robots and provide a new concept for developing next-generation bioactuators [22].

Compared with conventional rigid materials, hydrogels possess flexibility, regulable mechanical strengths, ion conductivity, transparency, ability to swell/shrink, and other unique properties that are suitable as candidates for building blocks for the fabrication of soft bioactuators/robots [6]. Notably, these properties also endow hydrogels with stimuli-responsive functions to adapt to physiochemical changes in the environment. Therefore, hydrogels are promising as materials for applications in 4D printing techniques [6]. Recently, stimuli-responsive polymer-based hydrogel systems can be used to develop various bioactuators/robots for different purposes through the selection of polymeric functional groups, the blending of heterogenous polymers, and the modification of polymers. For example, N-isopropyl acrylamide (NIPPAM) is a typical thermo-responsive polymer that can execute the sol-gel phase transition, swell, and expand the size of NIPPAM-based hydrogels. Therefore, Melocchi and co-workers printed NIPPAM-based structures by directly using two-photon laser writing and printing technology [23]. By controlling the power of a two-photon laser, the microstructures of the printed object with a crosslink gradient allow the object to transform its shape under the treatment of solvents with different polarities. Also, Gladman et al. developed an NIPPMA/Laponite/nano fibrillated cellulose (NFC) hybrid clay ink to fabricate 4D printed biomimetic actuators [15]. According to the orientation of the NFC in the printed fibers, the printed flower structure can fold and the petals of the flower can sway in the water after the swelling of the printed structures. In addition to the thermo-responsive property, Kang and co-workers developed an electro-responsive actuator by combing poly(3-sulfopropyl acrylate, potassium salt) (PSPA) and glycidyl methacrylated hyaluronic acid (GMHA) [24]. In their study, they demonstrated that adding GMHA significantly improved the mechanical properties of PSPA polymers and retained the electroactivity of PSPA polymers. Thus, the PSPA/GMHA hybrid hydrogel provided an ion movement in the hydrogel which caused an internal asymmetric ion distribution in the hydrogel after electrical stimulation. Subsequently, the asymmetric distribution behaviors generated an osmotic pressure difference at the cathode and anode sites of the hydrogel and further bent the hydrogel via a heterogeneous local swelling. Afterward, the phenomenon allowed an electroactuation mechanism to control the finger movement of a printed biomimetic PSPA/GMHA-based hand structure by regulating an alternative voltage. Moreover, another type of stimulation, magnetic stimulation, can achieve the untethered operation of soft actuators/robots via the 4D printing technique. Based on this concept, Zhang and co-workers combined a silicon elastomer and magnetic neodymium-iron-boron microparticles to print a soft and flexible biomimetic bionic butterfly and turtle [25]. The printed butterfly shows a flapping-swing motion, and the printed turtle can execute the crawl and swim behaviors under the control of a magnetic field. According to the results, Zhang and co-workers also printed a magnetic-responsive hand with a reprogrammable and controllable property that can play a rock-paper-scissors game by regulating the magnetization direction field [25].

Gelatin methacryloyl (GelMA) is a biocompatible, biodegradable, and chemically photo-crosslinkable polymer under UV light exposure, which has confirmed its printability for fabricating a versatile range of biomimetic constructs [26,27]. Despite reports confirming the printablity of GelMA inks, the challenging development of GelMA-based inks to directly print stimuli-responsive constructs is required to optimize their mechanical properties, printability, and actuation behaviors according to the characteristics of stimuli-responsive materials and trigger sources, such as temperature, pH, magnetic force, etc [21]. In our previous study, a printed GelMA hydrogel with homogeneously incorporated carbon nanotubes can move under an electrical stimulation field [21]. Therefore, the result indicates that GelMA polymers can act as a substrate to develop stimuli-responsive inks by mixing with other materials. Based on this experience, we selected the GelMA polymer as an ink candidate and used a direct-ink-writing (DIW) printing technique to develop *Mimosa pudica*-inspired printed constructs for soft bioactuator/robot applications. Specifically, we regulated the concentrations of ION and GelMA polymers to optimize the gelation behavior, mechanical properties, and magnetic-responsive actuation of crosslinked ION-GelMA hydrogels. Then, the rheological properties, printability, and biocompatibility of ION-GelMA inks were evaluated. Finally, the shape transformation behaviors were estimated by controlling the patterns of the constructs via a DIW printing technique. As a proof of concept, we used a photo-crosslinkable GelMA/iron oxide nanoparticle (ION) ink to provide the printed constructs with a magnetic-responsive function and controllable actuation behaviors, as well as propose a potential of ink for the 4D printing of soft bioactuators/robots.

## 2. Materials and Methods

### 2.1. Materials

Iron (II) chloride tetrahydrate (purity > 99.0%, product no. 44939), Iron (III) chloride hexahydrate (purity > 99.0%, product no. 31232), type A gelatin from porcine skin (Gel strength 300, product no. G2500), methacrylate anhydride (MA) (purity ≥ 94.0%, product no. 276685), 2-Hydroxy-4′-(2-hydroxyethoxy)-2-methylpropiophenone (Irgacure 2959) (purity ≥ 97.5%, product no. 410896), and alginic acid salt (low viscosity (4–12 cps), product no. A1112) were purchased from Sigma-Aldrich (St. Louis, MO, USA). Calcium chloride (CaCl_2_) (purity = 93.0%, product no. 12316, Alfa Aesar, Haverhill, MA, USA) was purchased from Thermo Fisher Scientific Inc. (Waltham, MA, USA)

### 2.2. Preparation of GelMA Polymers

The protocol for synthesizing GelMA polymers with the medium substitute degree followed our previous studies [28,29]. Briefly, 10% type A gelatin powders from porcine skin were added into Dulbecco’s phosphate-buffered saline (DPBS) at 50 °C and then stirred at 240 rpm until the gelatin powder dissolved completely. Subsequently, MA was gently added into the gelatin solution drop by drop, and the final concentration of MA was 1.25 *v*/*v*%. After 2 h of reaction at 50 °C under a low-speed stirring process, an equal volume of pre-warm DPBS was added into the gelatin/MA solution, followed by another stirring process for 10 min. Afterward, the mixture was moved to the dialysis membrane (Spectra/Por molecular porous membrane tubing, MWCO 12,000–14,000, Thermo Fisher Scientific Inc., Waltham, MA, USA) to execute a purification process. In the purification process, the GelMA mixture in the dialysis membrane was placed in distilled water (DI water) at 40 °C at 500 rpm, and the DI water was changed twice daily. After 5 days of dialysis, the purified GelMA polymers were moved to the centrifugal tubes and further stored at −80 °C. The GelMA polymers were obtained via a 4-day freeze-dried process to remove the DI water before experiments.

### 2.3. Synthesis of IONs

To synthesize IONs, we followed the previous studies and slightly modified the protocol of the co-precipitation method [30]. In short, an iron oxide precursor solution was prepared by mixing 8.94 g FeCl_2_·H_2_O and 18.3 g FeCl_3_·6H_2_O in 150 mL DI water under a stirring process. Subsequently, 5.8 M NaOH solution (50 mL) was slowly added drop by drop in the iron oxide precursor solution and reacted with a vigorous stirrer process. After 30 min of reaction at room temperature, the color of the iron oxide mixture changed from brown to black, and then the iron oxide precursor solution was dispersed by using an ultrasonic probe (1.5 min with a 3 s on/off pulse at a 20% amplitude, Q500 Sonicator, Qsonica L. L. C., Newtown, CT, USA). Furthermore, the black iron oxide solution was washed with DI water, and co-precipitated IONs were collected via a centrifugal procedure (10,000 rpm for 30 min). Then, the collected IONs were resuspended and dispersed in DI water and stocked at 4 °C. For use in experiments, the IONs were pre-treated in an ultrasonic procedure (1.5 min with a 3 s on/off pulse at 20% amplitude) for redispersion.

### 2.4. Identification of the Synthesized GelMA Polymer and ION

The Fourier transform infrared spectroscopy (FTIR) (PerkinElmer, Waltham, MA, USA) and multipurpose X-ray thin-film micro area diffractometer (XRD) (Bruker, Billerca, MA, USA) were used to identify the synthesized GelMA polymers and IONs. The wave range of FTIR conditions was 4000–500 cm^−1^. The scanning range and rate of XRD analysis were 5–10° at 2θ range and 2°/min. The size and surface charge of IONs were identified by using a Dynamic Light Scattering Nanoparticle Analyzer (DLS) (SC100, HORIBA, Kyoto, Japan) and Zetasizer (Malvern Panalytical, Malvern, Worcestershire, UK). The morphology of IONs and ION-GelMA hydrogels was observed by using a transmission electron microscope (TEM) (JEM-1400, JEOL, Tokyo, Japan) and a Schottky field emission scanning electron microscope (FE-SEM) (JSM-7800F, JEOL, Japan).

### 2.5. Preparation of ION-GelMA Hydrogels

5 mg, 10 mg, 15 mg, 30 mg, 50 mg, 75 mg, and 100 mg synthesized IONs were added in 1 mL DI water individually and then mixed 50 mg or 100 mg GelMA polymer, 10 mg alginic acid salt (1 *w*/*v*%), and 5 mg Irgacure 2959 (0.5 *w*/*v*%) to prepare 5 *w*/*v*% and 10 *w*/*v*% GelMA inks with the different ION concentrations. The mixture was placed at 50 °C for 30 min with a stirring process until the polymers and Irgacure 2959 dissolved completely. The dissolved ION-GelMA solutions were added into a poly(methyl methacrylate) (PMMA) cylinder mold (diameter = 8 mm and height = 4 mm) to fabricate a cylinder-shape ION-GelMA hydrogels by treating UV light (800 mW/cm^2^) for the 60 s. After photo-crosslinking, the ION-GelMA hydrogels were immersed in a 300 mM CaCl_2_ solution for 120 s to execute a secondary physical crosslink process and then gently removed from the mold. Afterward, the crosslinked hydrogels were used for subsequent testing.

### 2.6. Characterization of Mechanical Properties

The protocol for testing the compressive modulus of ION-GelMA hydrogels was according to our previous study [29]. Briefly, the compressive modulus of cylindrical ION-GelMA hydrogels was tested by using a texture analyzer (RapidTA, Horn Instruments Co., Ltd., Taoyuan, Taiwan) at 20% strain/min. The slope at the linear region, 0% to 10% strain, was selected to evaluate the compressive modulus.

### 2.7. Swelling Ratio and Mass Loss Testing

The protocols for swelling ratio and mass loss testing for GelMA and ION-GelMA hydrogels were according to our previous study [29]. In short, the photo-crosslinked cylinder-shaped GelMA and ION-GelMA hydrogels were treated with a freeze-dried process to obtain the freeze-dried hydrogels without water. Subsequently, the freeze-dried hydrogels were immersed in DPBS at 37 °C for 1, 3, and 7 days. Then, the hydrogels were collected, and the excess water was removed. Afterward, the weight of the swollen hydrogel was recorded (*W*_2_), and the swelling ratio was determined by using the weight of the swollen hydrogel to divide the dried weight of their initial freeze-dried hydrogel (*W*_1_).

$$Swelling\;ratio\left(\%\right)=\frac{W_{2}}{W_{1}}\times 100\%$$

To obtain the degradation behavior of photo-crosslinked GelMA and ION-GelMA hydrogels, the hydrogels were cultured in DPBS to evaluate their mass loss ratio. Similar to the protocol of swelling ratio testing, the weight of initial freeze-dried hydrogels was collected (*W_I_*). Subsequently, the initial freeze-dried hydrogels were soaked in DPBS and then incubated at 37 °C for 1, 3, and 7 days. Afterward, the DPBS was gently removed, and the remaining hydrogels were treated with another freeze-dried process. The dried weight of the remaining hydrogels was recorded (*W_D_*). Then, the mass loss behavior was calculated by the following equation.

$$Mass\;Loss\left(\%\right)=\frac{W_{I}-W_{D}}{W_{I}}\times 100\%$$

### 2.8. Characterization of Rheological Properties

The rheological property was used to evaluate the fluidic behaviors of ink. The ION-GelMA ink was tested by using a Discovery HR20 rheometer (TA Instruments, New Castle, DE, USA). The relationship between viscosity, and strain% was evaluated under a linear ramp shear rate (1 to 100 1/s) and the response of the ION-GelMA ink was determined over a continuous flow.

### 2.9. Printability of ION-GelMA Ink

The ION-GelMA ink was loaded in 5 mL syringes with 24G needles and then locked on an extruder of a commercial 3-axis dispensing robot (GB-5203, Ganbow Technology, New Taipei City, Taiwan). The printing conditions were set as follows: Nozzle moving rate = 5 mm/s and extrusion pressure = 1.5~2.0 psi. Subsequently, the tip of the needle was moved to the designed position, which represented the initial point for all the printing structures.

To optimize the ideal ink condition, the printing resolution of ION-GelMA inks was evaluated by using a semi-quantification method from a previous study [31]. Briefly, an ideal condition could offer a printability (Pr value) that the extruded filaments had a constant width and smooth surface in three dimensions, so the interconnected square holes of printed constructs were regular and without deformation behavior when the Pr value = 1. A Pr value < 1 indicated that an unsuited condition could cause the extruded filaments to have a more liquid-like state and induce the formation of circular holes. In contrast, the extruded filaments with an over-gelation behavior could cause the irregular shapes of the interconnected holes when the Pr value > 1 Therefore, the previous study defined the printing resolution of the extruded filaments by using a printability formula [31].

$$\mathrm{Pr}=\frac{\pi }{4}\times \frac{1}{C}=\frac{L^{2}}{16A}$$

Pr: Printability
*C*: Circularity of an enclosed area
*L*: Perimeter
*A*: Area

### 2.10. Fabrication of Mimosa pudica-Like Constructs by a Mold Casting Method and DIW Printing Technology

To fabricate mold cast *Mimosa pudica*-like constructs, the pure GelMA and the ION-GelMA inks were individually poured into the vascular tissue part and pinnule leaf parts of a PMMA mold (Overall size = 2 cm × 2 cm, the thickness = 3 mm). After 60 s of UV treatment, the casting construct was immersed in a 300 mM CaCl_2_ solution for another 120 s of ionic crosslinking. Afterward, the casting constructs were gently removed from the PMMA mold.

To fabricate printed *Mimosa pudica*-like constructs, we used a commercial 3-axis dispensing robot with dual extruders (GB-5203, Ganbow Technology, New Taipei City, Taiwan) to execute the printing of pure GelMA and ION-GelMA inks individually. The printing conditions were set as follows: Nozzle moving rate = 5 mm/s and extrusion pressure = 1.5–2.0 psi. Subsequently, the tip of the needle was moved to the designed position, which represented the initial point for the printing of *Mimosa pudica*-like constructs. In the printing procedure, the pure GelMA ink first printed a vascular tissue part. Subsequently, the ION-GelMA ink was used to print the pinnule leaf part to connect the printed vascular tissue part to obtain a DIW printed *Mimosa pudica*-like construct (overall size = 2 cm × 2 cm, 5 layers, thickness ≈ 1.5 mm). After 60 s of UV treatment, the printed construct was further immersed in a calcium chloride solution for another 120 s of ionic crosslinking.

### 2.11. Statistical Analysis

We evaluated the statistical analysis in this study by using the SAS analytic software version 9.4 (SAS Institute Inc., Cary, NC, USA). The analytic method was one-way ANOVA, and the following post hoc test was Tukey’s test to compare data (* *p* < 0.05, ** *p* < 0.01).

## 3. Results and Discussion

### 3.1. Preparation and Optimization of ION-GelMA Hydrogels

**Figure 1:**
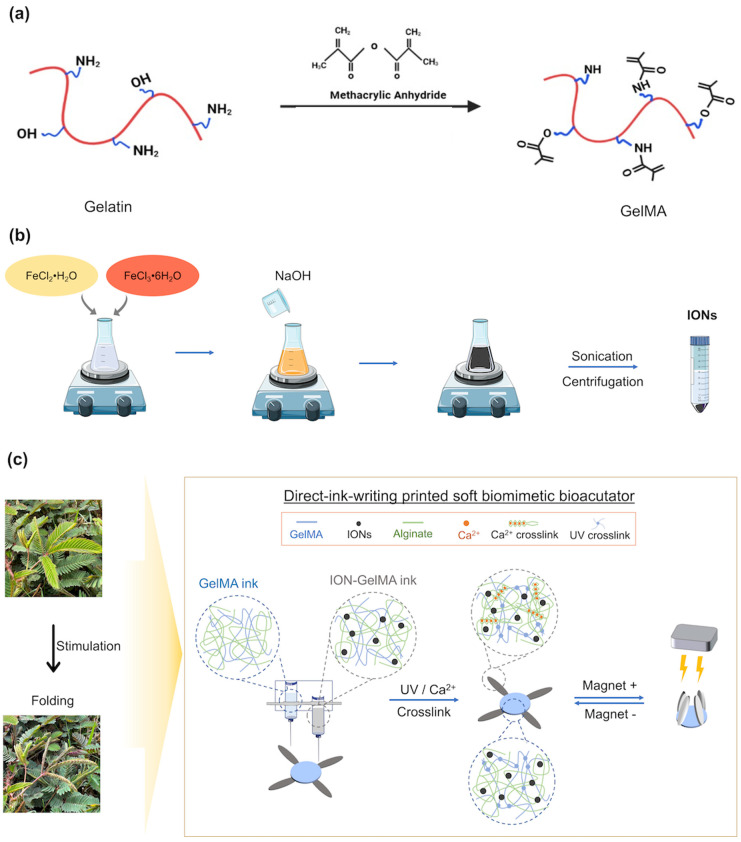
Schematic illustration of ION-GelMA inks for applications in biomimetic soft bioactuators/robots. Representation of the synthesis of (**a**) GelMA and (**b**) IONs, and (**c**) the fabrication of printed biomimetic *mimosa pudica*-like magnetic-responsive bioacutaors.

Soft bioactuators or robots are considered novel tools that can mimic bio-like biological features to design objects with the desired functions for different purposes. Magnetic force is a force at a distance that contributes a new strategy for the untethered operation of the movement of soft micro- and nano-robots. Under a controllable magnetic field, magnetic-responsive materials provide a feasible way to convert magnetic energy into locomotion to drive soft bioactuators/robots. Therefore, magnetic-responsive bioactuators or robots have become increasingly attractive for designing specific functions for translation into clinical practice and future biomedical applications, such as biosensors, diagnosis, microsurgery, and targeted drug delivery. To create biocompatible soft bioactuators, previous studies have used photo-crosslinkable GelMA polymers to print the soft microswimmers with a helical structures via two-photon polymerization [32,33]. Then, the printed helical microswimmers were immersed in an ION solution to coat a thin ION-based layer. Afterward, the coated layer can endow the printed micro-swimmers with a magnetic-responsive function, which can be driven to move under a magnetic field. Unlike previous studies [32,33], we developed a photo-crosslinkable injectable ION-GelMA ink to fabricate magnetic-responsive soft bioactuators in this study (Figure 1). According to our design, the gelatin polymers were modified by using methacrylic anhydride. After modification, the methacryloyl group allowed GelMA polymers to have a photo-crosslinkable ability (Figure 1a). The IONs were synthesized through a co-precipitation method, and the synthesized IONs possessed a superparamagnetic ability (Figure 1b) [30]. Subsequently, the IONs endowed the ION-GelMA ink with a magnetic-responsive ability when the IONs were in the presence of the ink. Additionally, we also added alginic acid salt to the ION-GelMA ink. The alginic acid salt can interact with calcium ions and contribute to a secondary physical crosslink interaction for the formation of printed structures after printing the structure. The novelty of this design is that we first combine the photo-crosslinkable GelMA and IONs into a hybrid ink for use in the DIW printing technique. *Mimosa pudica* is a mechanically responsive plant that can convert the pressure to the motion of leaves [34,35]. When the leaves receive pressure, the cells can redistribute the water in the pulvinus which results in asymmetric turgor in the extensor and flexor sides of the pulvinus. Afterward, the different turgor pressure between the extensor and flexor sides further induces the bending of the pulvinus. Inspired by the actuation of *Mimosa pudica*, we wanted to design a soft bioactuator that can convert magnetic force to a driving force for the actuation of printed constructs. Therefore, we printed a *Mimosa pudica*-like biomimetic soft bioactuator to evaluate the potential of ION-GelMA for applications in soft bioactuators/robots. Notably, we hypothesized that the ION-GelMA ink can provide the dual crosslinking mechanisms to enforce the mechanical properties of printed structures and further offers a magnetic-responsive function to drive the movement of printed structures (Figure 1c).

**Figure 2:**
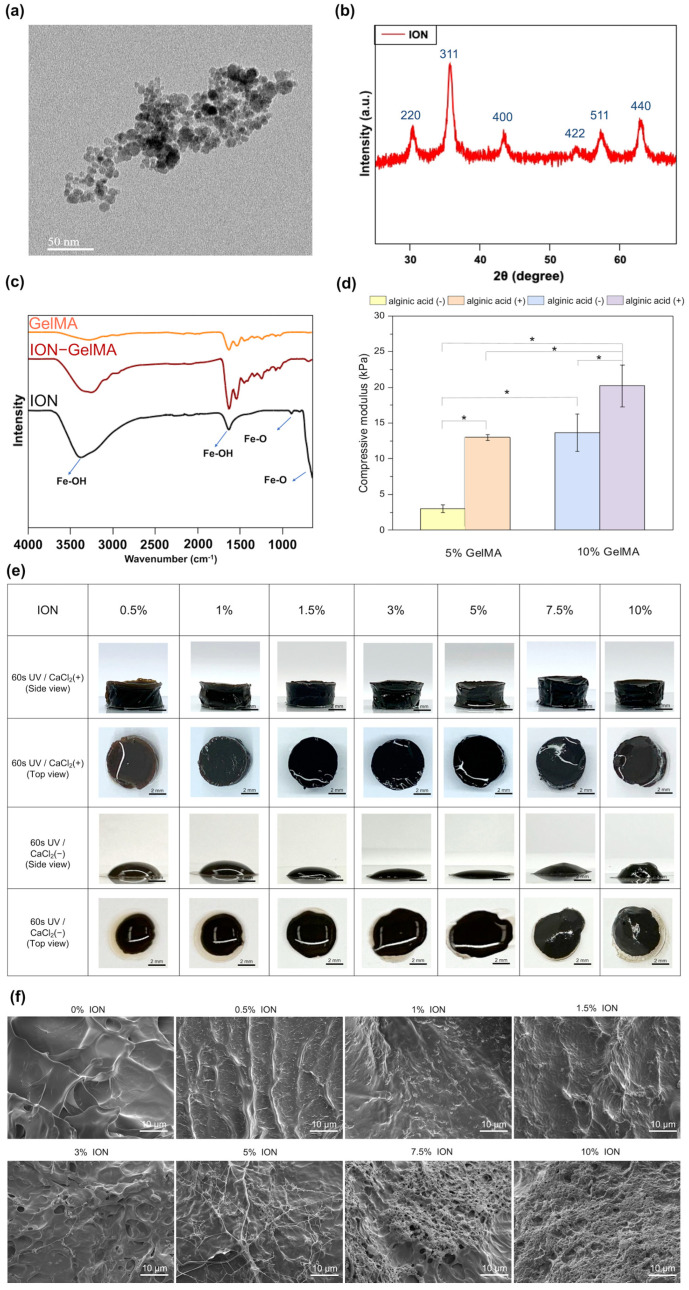
Characterization of IONs and ION−GelMA hydrogels. (**a**) The TEM image shows the IONs with a nanoscale size. (**b**) The XRD spectrum confirmed the synthesis of IONs with a typical ION X-ray diffraction pattern. (**c**) The FTIR spectrum shows the characteristic peaks of the functional groups of GelMA polymers, ION−GelMA inks, and IONs. (**d**) The compressive modulus of GelMA hydrogels in the absence or presence of alginic acid after UV crosslink. * denotes a significant difference (*p* < 0.05) between the two groups. (**e**) The optical images show the ION−GelMA hydrogels with different ION concentrations after UV crosslink and the treatment of calcium chloride solution. (**f**) The SEM images indicate the ION morphology in the cross-section of ION−GelMA hydrogels.

In the present study, FeCl_2_·H_2_O and FeCl_3_·6H_2_O were used to synthesize IONs via the co-precipitation method. To characterize the synthesized IONs, we first aimed to analyze the size and morphology of the IONs. The TEM image indicated that the size of synthesized IONs was nearly 7.5 ± 2.0 nm and the molecular interactions among the IONs caused a slight aggregation morphology (Figure 2a). Furthermore, we also identified the structural pattern of IONs by using XRD. Figure 2b shows that the XRD spectrum has six peaks at 2θ at 30.1, 35.5, 42.6, 53.6, 57.0 and 62.8, which matched the (2 2 0), (3 1 1), (4 0 0), (4 2 2), (5 1 1), and (4 4 0) planes. The result is close to a standard γ-Fe_2_O_3_ reflection [36]. Next, the IONs mixed with GelMA after treating with UV light were identified by using the FTIR analysis to analyze the interactions between GelMA polymers and IONs (Figure 2c). First, the typical peaks of pure GelMA polymers related to the C-O, C–N, C-O-C, and amine bonds in the FTIR spectrum were significantly observed at 1630, 1531, 1040, and 3280 cm^−1^. The peaks of the Fe-O bond of pure IONs were observed at 650 and 890 cm^−1^ [37]. In addition, the strong peaks of the Fe-OH bonds in the pure ION group existed at 3384 cm^−1^, which indicated that the surface of synthesized IONs contained hydroxy groups [37]. These hydroxy groups on the ION surface endow IONs with a negative surface charge (−39.2 ± 0.72 mV) and offer hydrogen bonding to cause the aggregation of the IONs (Figure 2a) [38]. As shown in Supporting Figure S1, the hydrodynamic diameter of the aggregated IONs is 329.2 nm. After encapsulating the IONs in the GelMA, the ION-related peaks can also be observed in the ION-GelMA groups, especially the peaks at 1630 and 3384 cm^−1^. The other related characteristic peaks of the IONs were also enhanced following the increase in ION concentration, (Supporting Figure S2). In addition, a new peak at 690 cm^−1^ was produced because small shifts were associated with the interactions among the relevant Fe-O bonds, internal molecular water, and GelMA polymeric chains during the mixing process. Furthermore, to prepare the hydrogel with dual crosslink mechanisms for the printed structures (i.e., chemical and physical crosslink interactions), 1% alginic acid polymers were mixed with 5% and 10% GelMA polymers. After crosslinking, compared with the hydrogel without the alginic acid, the compressive modulus of the GelMA hydrogel in the presence of alginic acid was significantly increased, and the strength that the hydrogels could achieve was nearly doubled. Muscle-inspired bioactuators have been used for a wide range of applications. Therefore, we selected the 10% GelMA polymer mixed with 1% alginic acid polymer as the ink composition for subsequent testing because the modulus of muscle is in the range of 8–15 kPa [39]. Next, to evaluate the effect of IONs on the formation of hydrogel, IONs were mixed with GelMA and alginic acid polymers to prepare the ION-GelMA ink with different concentrations of IONs. Then, the ION-GelMA inks were poured into a cylindrical mold and crosslinked by treatment with 60 s of UV light radiation at an 800 mW/cm^2^ intensity. However, the IONs can absorb the UV energy to decrease the generation of free radicals and cause a slight collapse of ION-GelMA hydrogels (Figure 2e). Although the hydrogel containing 10% IONs can form a gel-type construct because of the internal interaction between the IONs and gelatin chains, the structure of the hydrogel was an irregular shape after the treatment of UV light. Therefore, the UV-treated hydrogels were further immersed in a 300 mM calcium chloride solution because the presence of alginic acid in the ink provided a physical interaction with calcium ions to maintain the hydrogel formation via a secondary physical crosslink process [40]. Compared to the groups without the treatment of calcium chloride, the hydrogels showed a completely cylindrical shape after the secondary crosslink process. Subsequently, we used SEM to observe the microstructure of the ION-GelMA hydrogels (Figure 2f). Through the cross-section SEM images, the morphology of the GelMA and ION-GelMA hydrogels at low ION concentrations (i.e., 0.5–1%) shows no significant differences. The morphology of the IONs in ION-GelMA hydrogels showed the gradual appearance of ION aggregation following the ION concentration increasing from 1.5% to 5%, as well as a fiber network forming from the increase in internal molecular interactions between the IONs and polymeric chains. At the same time, a similar trend of ION aggregation could be found in 1.5–5% ION-GelMA hydrogels when using cryosection technology to identify the overall hydrogel morphology (Supporting Figure S3). Furthermore, Figure 2f shows the behaviors of an obvious ION-polymeric chain fiber network and strong aggregation when the ION concentration reaches 7.5% and 10%. Also, a high ION concentration at 10% with strong internal ionic interactions can induce slight shrinkage and the formation of a pore in the hydrogels (Supporting Figure S3). Based on the results from Figure 2f and Figure S3, the microstructure of ION-GelMA hydrogels shows their morphology which are the IONs distributed in the GelMA hydrogel and the GelMA polymers covering the IONs.

**Figure 3:**
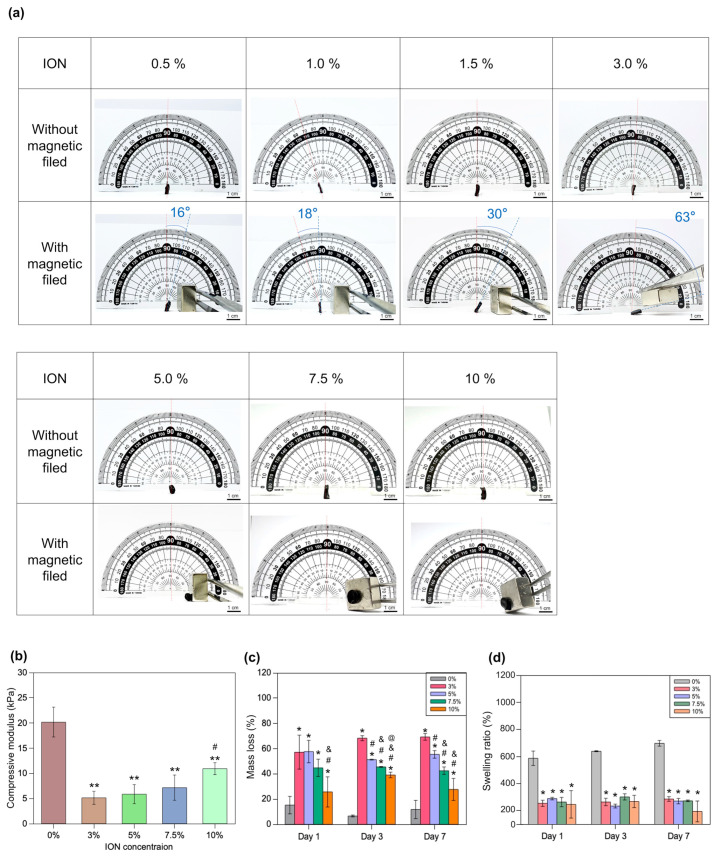
(**a**) Optical images of the magnetic field-induced movement process of ION-GelMA hydrogels with different ION concentrations. (**b**–**d**) The compressive modulus, mass loss percentage, and swelling ability of ION-GelMA hydrogels with different ION concentrations after 60 s of UV treatment. (*: comparison with 0% group, *p* < 0.05; **: comparison with 0% group, *p* < 0.01; #: comparison with 3% group, *p* < 0.05; &: comparison with 5% group, *p* < 0.05; @: comparison with 7.5% group, *p* < 0.05).

Afterward, the ION-GelMA hydrogels were also estimated for their magnetic properties, mechanical strength, swelling, and degradation behaviors (Figure 3). First, we evaluated the effect of ION concentration on the locomotion of ION-GelMA hydrogels by applying a magnet on a horizontal plane (Figure 3a). Here, a commercial NdFeB magnet (15 mm × 15 mm × 10 mm, ND-35, surface magnesium ~4400 G ± 10%, Magtech Magnetic Product Corp., Taiwan) was used to provide a magnetic force. Under a magnetic field and a fixed distance (~5 mm) between the hydrogels and the magnet, we investigated the magnetic-responsive ability of hydrogels containing different ION concentrations. When hydrogels were mixed with low ION concentrations (i.e., 0.5% and 1%), the hydrogels started to bend, and the bending angles were 16 and 18 degrees. Following the increase in ION concentration, the bending angles that 1.5% and 3% ION-GelMA hydrogels could achieve were 30 and 63 degrees because of the increase in the interaction between the IONs and the magnet. Interestingly, once the ION concentration was more than 5%, the high ION concentration could endow the ION-GelMA hydrogels with a strong interaction with the magnet, resulting in the hydrogels being attracted to the magnet, leaving their original location and moving toward the magnet. Based on the results, we selected 3%, 5%, 7.5%, and 10% ION-GelMA hydrogels for subsequent experiments because these ION concentrations can provide the hydrogel with significant movement. Furthermore, previous studies indicated that IONs could improve the mechanical properties of GelMA-based hydrogels [32,33]. However, we observed that the mechanical strength of ION-GelMA hydrogels was lower than that of a pure GelMA hydrogel, and the average compressive modulus of all ION-GelMA hydrogels was 5 kPa to 10 kPa (Figure 3b). Unlike the procedures in previous studies [32,33], we mixed IONs and GelMA as an ink in our study. Then, the ION-GelMA ink was used to directly fabricate the constructs, and the constructs were crosslinked under a UV light. IONs can absorb UV light energy [41,42], which may interrupt the crosslinking of GelMA chains. For example, in another previous study in which Tognato and co-workers also mixed PEG-modified IONs and GelMA as a solution to fabricate ION-based hydrogels [43], the compressive modulus of the ION-based hydrogels slightly decreased. Therefore, a similar phenomenon can be observed in our study. Moreover, despite the decrease in the compressive modulus in the ION-GelMA hydrogels in our study, Figure 3b still shows an increase in the compressive modulus of ION-GelMA hydrogels following an increase in the ION concentration (3% to 10%). This result indeed confirms that the addition of magnetic IONs can improve the mechanical properties of hydrogels. Additionally, we also found that the compressive modulus of ION-GelMA hydrogels could be slightly increased if the UV exposure time was increased to 120 s (Supporting Figure S4), but no significant increase at the high ION concentration groups was observed. The phenomena could be attributed to the ION absorbing the UV light energy and interrupting the crosslinking of GelMA chains [41,42]. Figure 3c,d show the mass loss and swelling behaviors of ION-GelMA. Compared with the pure GelMA group, we observed that the mass loss of ION-GelMA hydrogels was higher than the pure GelMA hydrogel, and the swelling ratio of hydrogels was lower than pure GelMA after 1, 3, and 7 days of incubation in DPBS solution. Due to the effect of IONs, low ION concentrations can cause the inefficient crosslinking of GelMA, and 3% and 5% GelMA hydrogels were observed to exhibit a high mass loss behavior. Notably, 7.5% and 10% ION-GelMA hydrogels also exhibited inefficient internal crosslinking, but high ION concentrations in the hydrogels may contribute to ionic/electrostatic interactions and hydrogen bonding with the functional groups of the GelMA chain, such as amine or carboxyl groups [44]. Therefore, these interactions can enforce the remaining crosslinked GelMA parts and reduce the mass loss and swelling ratio of ION-GelMA.

### 3.2. Evaluation of the Injectability, Printability, and In Vitro Cytotoxicity of ION-GelMA Hydrogels

#### 3.2.1. The Injectability of ION-GelMA Hydrogels

**Figure 4:**
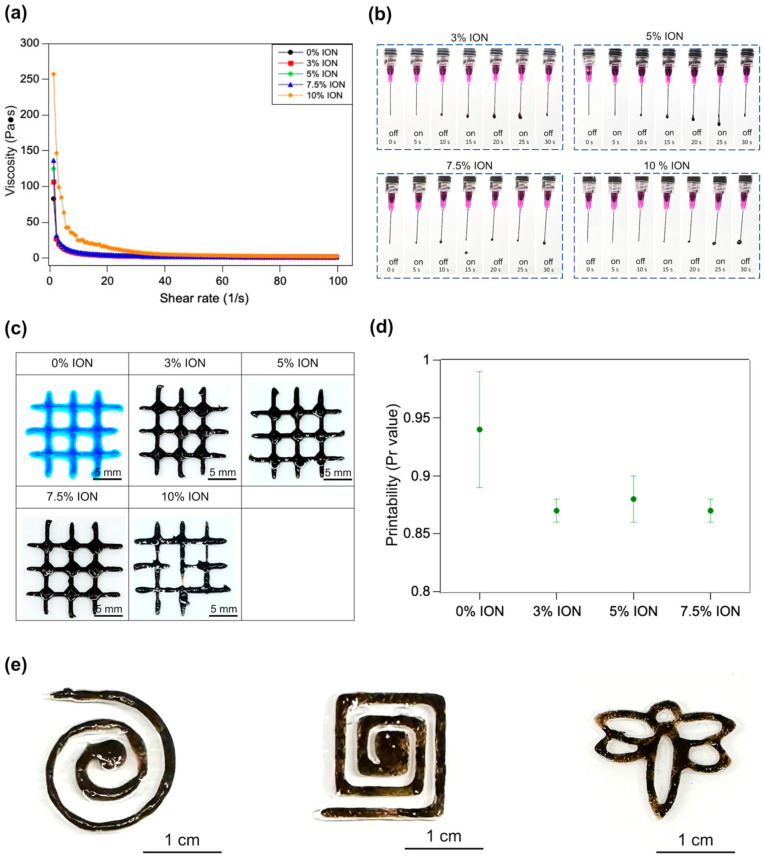
Evaluation of injectability and printability of ION-GelMA inks. (**a**) The effect of ION concentrations on the shear-thinning behavior of ION-GelMA inks. (**b**) The injectability testing of ION-GelMA inks with different ION concentrations shows the inks with a rapid gel-fluid transition behavior. (**c**) The optical images and (**d**) the semi-quantified printability of a printed grid pattern made from ION-GelMA inks. (**e**) The printed patterns (i.e., spiral, square-spiral, and dragonfly) of 5% ION-GelMA ink through an extrusion 3D printer.

In order to evaluate the injectability and printability of the ION-GelMA inks, the rheological properties of the inks were estimated using a rheometer. Firstly, we investigated the effect of the IONs on the fluidic behavior of ION-GelMA inks through a linearly ramped shear rate model. The apparent viscosity of GelMA inks with different ION concentrations significantly decreased with an increasing shear rate, indicating that the ION-GelMA and pure GelMA inks exhibited non-Newtonian fluid and shear-thinning behaviors. In a comparison with the pure GelMA ink, the presence of IONs promoted a physical crosslinking reaction, increased the viscosity of the inks, and caused a slow-flowing behavior that may contribute to the injectability of the ION-GelMA inks (Figure 4a). Additionally, we further evaluated the injectability of ION-GelMA inks by directly observing the inks as they were injected out from a nozzle. In the 3% and 5% ION-GelMA inks groups, the inks extruded from the nozzle tip exhibited a sticky liquid morphology, especially the 5% ION-GelMA ink (Figure 4b). In the other ION concentration groups, the stronger ionic/electrostatic interactions caused the 7% and 10% ION-GelMA ink to easily form a droplet morphology. In particular, the 10% ION group showed that the ink is difficult to extrude from the nozzle, indicating that 10% IONs in the GelMA ink can induce the aggregation of metal ions and GelMA chains, lead to over-gelation, and appear as having a dense slurry morphology (Figure 2f and Figure 4b).

#### 3.2.2. The Printability of ION-GelMA Hydrogels

Furthermore, the printability of the ION-GelMA inks was evaluated by using a semi-quantitative method to confirm the Pr value (Figure 4c,d). Our previous study has shown that substrates with different contact angles, such as glass (~20°) or PMMA (~80°) can slightly affect the printed structures [45]. In this study, the inks were water-based solutions, and a highly hydrophilic glass substrate could cause the printed fiber to spread on the substrate. Therefore, we selected a relatively hydrophobic PMMA plate as the substrate to prevent the spread of the printed fibers. Based on the same substrate, compared to the pure GelMA ink, the inks with less than 10% IONs could be printed and maintain the regular shape of the fiber and interconnected channels as a square (Figure 4c). Figure 4d shows that the Pr values of the 0%, 3%, 5%, and 7.5% ION-GelMA inks are 0.94 ± 0.05, 0.87 ± 0.01, 0.88 ± 0.02, and 0.87 ± 0.01. Although the Pr values of the inks with IONs were slightly lower, there was no significant effect on the printability of the inks. However, the printed pattern using the 10% ION-GelMA ink shows a noncontinuous and non-uniform interconnected square morphology. A possible reason may be attributed to high internal molecular interactions causing the inks to present a low-smooth extrusion (Figure 4c,d).

#### 3.2.3. The Cytotoxicity of ION-GelMA Hydrogels

Based on the results from the printability experiments, we further evaluated the in vitro cytotoxicity of the 3%, 5%, and 7.5% ION-GelMA groups following the ISO 10993 [46] protocol used in our previous study [47]. The results showed that the the 3%, 5%, and 7.5% ION-GelMA groups can achieve a cell viability of more than 90% and have no significant differences from cells cultured on TCPS (control group) (Supporting Figure S5). Therefore, the 3%, 5%, and 7.5% ION-GelMA inks were selected for use in further experiments in this study. Here, we used the 5% ION-GelMA as a sample to print spiral, square-spiral, and dragonfly patterns, and to evaluate the potential application in a cell culture. The printing results indicated that the ION-GelMA ink has a potential in printing applications (Figure 4e). In addition, the F-actin fluorescent image shows that a muscle cell line, C2C12, can grow, align, and have a well-extension morphology on the surface of a printed 5% ION-GelMA grid structure (Supporting Figure S6). The results confirmed that the ION-GelMA construct has a potential for application in the cell culture or bioactuator fields.

**Figure 5:**
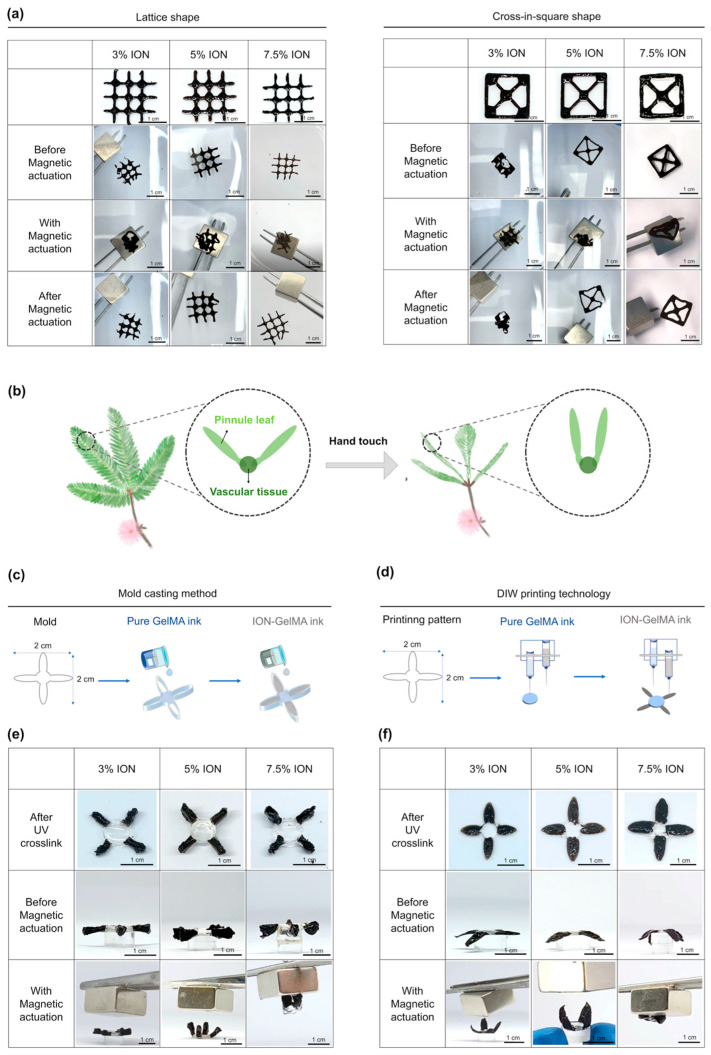
Evaluation of the magnetic-responsive actuation behavior of printed ION-GelMA constructs. (**a**) The optical images of printed 2D ION-GelMA lattice and cross-in-square shapes under a magnetic actuation process show the shapes with a transformation ability. (**b**) The bioactuation mechanism of *Mimosa pudica*. The schematic figures of the fabrication of ION-GelMA *Mimosa pudica*-like constructs through the (**c**) mold-casting method and (**d**) DIW printing technology. The optical images of (**e**) mold-cast and (**f**) printed ION-GelMA biomimetic *Mimosa pudica*-like constructs under a magnetic actuation process indicated the *Mimosa pudica*-like constructs with a magnetic-responsive actuation behavior. All scale bar = 1 cm.

Next, we printed a one-layered 2D lattice and cross-in-square patterns to compare the actuation behaviors of the 3%, 5%, and 7.5% ION-GelMA inks. The printed lattice structure in the 3% ION-GelMA group started to aggregate under a magnet as a trigger, and the structure could return to its original shape upon removal of the magnet. However, the 3% ION-based printed structure was slightly damaged after the magnetic stimulation. Additionally, the printed cross-in-square structure using the 3% ION-GelMAink could not be maintained before the magnetic actuation because the structure was weak (Figure 5a). Although the printed cross-in-square structure via the 3% ION-GelMAink could transform and aggregate when the magnet closed, the structure exhibited an irreversible morphology (Figure 5a). In the 5% ION-GelMA ink group, both the printed lattice and cross-in-square structures exhibited reversible transformation behavior because the structures could aggregate under magnetic actuation and return to their original structures without magnetic stimulation. Similar to the 5% ION-GelMA ink group, the printed structures made from 7% ION-GelMA ink also showed a magnetic-responsive actuation behavior. Compared with the cross-in-square structure in the 5% ION-GelMAink group, the strong internal molecular interaction of the 7% ION-GelMAink restricted the movement of the printed fibers and then decreased the actuation ability of the printed structure. Based on the results, the irreversible actuation and damage behaviors of the printed structures in the 3% ION group may be attributed to the low ION concentration and low ionic-polymeric chain interactions (Figure 2e and Figure 3a). In contrast, the ION concentrations of more than 3% could endow the printed structure with a strong strength to provide a reversible actuation behavior and prevent damage after magnetic stimulation (Figure 2e, Figure 3a and Figure 5a), but the stronger ionic-polymeric chains interactions in the 7% ION-GelMAink may limit the movement of the printed structure. According to these results, the 5% ION-GelMA ink may be a candidate for fabricating soft bioactuators/robots.

Moreover, in this study, we utilized ION-GelMA inks to validate the suitability of these inks for the fabrication of soft bioactuators/robots. Inspired by the folding up of the compound leaves of *Mimosa pudica* upon hand touching, we fabricated *Mimosa pudica*-like constructs with vascular tissue and pinnule leaf parts by using a mold casting method and DIW printing technology (Figure 5b). First, we employed pure GelMA ink to create a joint-like structure as the vascular tissue of *Mimosa pudica*. Subsequently, ION-GelMA inks were used to make the magnetic-responsive moveable structure for emulating the pinnule leaf of *Mimosa pudica*. The joint-like and moveable pinnule structures were assembled using the mold casting method or DIW printing technology. We hypothesized that the design of the ION-GelMA pinnule leaves and pure GelMA vascular tissue would constitute a *Mimosa pudica*-like structure, and that the pinnule leaf part could be stimulated magnetically to induce folding. Figure 5c,d indicate that the 3%, 5%, and 7% ION-GelMA inks could be fabricated into *Mimosa pudica*-like constructs by both using the mold casting method and DIW printing technology.

As shown in the results of the mold casting group (Figure 5c), the 3% ION concentration was insufficient to generate a magnetic interaction with the magnet to drive the thick pinnule structure, so the pinnule structure showed inadequate foldability behavior. Compared with the 3% ION group, the pinnule leaf part in the 5% ION-GelMA group bent inward and presented a proper fold under the magnetic force (Figure 5e). Afterward, the folding pinnule leaf part could return to its original state through the inherent elasticity of the GelMA, indicating that the 5% ION-GelMA ink had a reversible transformation ability. In addition, although the pinnule structure could be driven and moved by the magnetic force, the 7% ION concentration offered a strong magnetic-responsive effect that caused the whole mold-cast construct to be directly attracted to the magnet. The result presented the mold-cast 7% ION-GelMAconstruct with an over-folding behavior, and its transformation was dissimilar to the movement of *Mimosa pudica*. Therefore, 5% ION-GelMA ink might be adequate for the fabrication of soft bioactuators/robots. Despite the fact that the ION-GelMA constructs made using the mold casting method show potential for application in soft bioacutators/robots, in our case, we also observed that the constructs from the mold casting method exhibited rough and non-uniform phenomena and thick structures (thickness = 3 mm) that may limit the movement of the construct. Compared with the mold casting method, DIW printing technology was demonstrated to provide a higher degree of tolerance [48]. Therefore, we evaluated the movement of *Mimosa pudica*-like magnetic-responsive constructs made using DIW printing technology (Figure 5d). Figure 5f shows that the effect of the ION concentrations on the printed constructs was similar to the results of the mold casting method. Although the pinnule leaf movement of the printed 3% ION-GelMA construct was more obvious than the mold casting constructs, an insufficient movement of the pinnule leaves was observed. Compared with the 5% ION-GelMA constructs from the mold casting method (Figure 5e), the printed 5% ION-GelMA constructs were more refined and thinner (thickness = 1.5 mm) (Figure 5f), and the movement of the printed construct provided a more biomimetic transformation shape that was close to the movement of the pinnule leaf. Furthermore, the printed 7% ION-GelMAconstruct still had an over-folding behavior, indicating that the 7% ION concentration was unsuitable as the condition for use in our case.

Based on the results, the obvious advances of this study show that GelMA polymers can provide soft bioactuators/robots with tunable mechanical properties by controlling the UV exposure time compared with conventional hard elastomers [25]. Using ION-GelMA inks for DIW printing allows for the direct printing of magnetic-responsive constructs in a one-step fabrication procedure without the need for another coating process to load IONs onto the surfaces of the construct [32,33]. In addition, unlike the PEG-modified ION-GelMA ink used in another study [43], GelMA and alginate polymers as the composition of inks contribute a dual crosslink mechanism that can prevent the UV energy absorption effect of IONs on the formation of photocrosslinked ION-GelMA constructs and provide a rapid crosslinking time for fabricating soft bioactuators/robots using a UV light. Furthermore, compared with other magnetic-responsive hydrogel-based bioactuators/robots, the soft constructs printed using our design can be actuated in an air phase without any liquid as a medium under a magnetic field. Therefore, the ION-GelMA inks developed in this study were capable of DIW printing and contributed potential applications for making magnetic-responsive soft bioacutators/robots under appropriate conditions. Progress has been made in magnetic-responsive soft bioacutators/robots in the past decade. However, several challenges or limitations remain in the strategies for the design, fabrication, and actuation approaches in applications. For example, surface modification technology has been widely applied to improve the dispersity and stability of IONs in hydrogels, but the biodistribution and bioaccumulation of modified IONs need more toxicological evaluation to define their toxicity clearly [49,50]. In addition, to ensure the biocompatibility and nontoxicity of IONs in hydrogel- or cell-based soft bioactuators/robots, coating IONs with biomolecules provides an efficient strategy to enhance biocompatibility and has been approved by the US Food and Drug Administration [50]. Furthermore, compared with conventional materials, the costly process of precisely controlling the dispersity, shapes, and size of IONs constitutes a challenge in the scalability of magnetic-responsive soft bioactuators/robots because the mechanical properties, release profiles, safety, and scale-up of the IONs are not easily understood and predicted in large-scale production processes [51]. Recently, combining magnetic-responsive IONs with materials possessing other driving forces, such as chemistry, temperature, electric field, and others [52,53,54,55], has become one of the strategies for solving these challenges. In this design, hybrid properties enable the improvement of the mechanical durability, stability, and environmental adaptability of magnetic-responsive soft bioactuators/robots in a scale-up process [56] and are expected to bring broader future biomedical applications of magnetic-responsive soft bioactuators/robots.

## 4. Conclusions

In this study, we developed a photo-crosslinkable hybrid ink with magnetic-responsive properties based on IONs and bioactive GelMA polymers for fabricating soft bioactuators/robots. The magnetic-responsive properties of IONs and photo-crosslinkable GelMA enable the rapid and precise modulation of various properties, including mechanical strength, magnetic-responsive ability, degradation, swelling, and printability. Inspired by the movement behavior of natural creatures, we used ION-GelMA ink to print a magnetic-responsive construct as a biomimetic magnetic actuator that mimics the movement of *Mimosa pudica* in response to stimulation. This study demonstrates the novelty and successful application of ION-GelMA inks in magnetic actuation, which offers a promising strategy for 4D printing technology applied in broad applications in bionics, soft bioactuators/robotics, intelligent fabrication, and other fields.

## Data Availability

Data are contained within the article and Supplementary Materials.

## Supplementary Materials

The following supporting information can be downloaded at: https://www.mdpi.com/article/10.3390/polym16010025/s1. Figure S1: The hydrodynamic diameter of IONs was measured by using DLS size measurement; Figure S2: The FTIR spectrum of ION-GelMA inks with different ION concentrations; Figure S3: The optical images of ION distribution in the cryosection of GelMA and ION-GelMA hydrogels; Figure S4: The compressive modulus of ION-GelMA hydrogels with different ION concentrations. (*: comparison with 0% group, *: *p* < 0.05; Figure S5: The cell viability of ION-GelMA hydrogels; Figure S6: The cell morphology of C2C12 cells were cultured on the surface of printed 5% ION-GelMA structure after 7 days of incubation).

## References

[1] Acome E., Mitchell S.K., Morrissey T.G., Emmett M.B., Benjamin C., King M., Radakovitz M., Keplinger C. (2018). Hydraulically amplified self-healing electrostatic actuators with muscle-like performance. Science.

[2] Li M., Pal A., Aghakhani A., Pena-Francesch A., Sitti M. (2022). Soft actuators for real-world applications. Nat. Rev. Mater..

[3] Kaynak M., Dirix P., Sakar M.S. (2020). Addressable Acoustic Actuation of 3D Printed Soft Robotic Microsystems. Adv. Sci..

[4] Kang B.B., Choi H., Lee H., Cho K.J. (2019). Exo-Glove Poly II: A Polymer-Based Soft Wearable Robot for the Hand with a Tendon-Driven Actuation System. Soft Robot.

[5] Cianchetti M., Laschi C., Menciassi A., Dario P. (2018). Biomedical applications of soft robotics. Nat. Rev. Mater..

[6] Shi Q., Liu H., Tang D.D., Li Y.H., Li X.J., Xu F. (2019). Bioactuators based on stimulus-responsive hydrogels and their emerging biomedical applications. NPG Asia Mater..

[7] Mirvakili S.M., Hunter I.W. (2018). Artificial Muscles: Mechanisms, Applications, and Challenges. Adv. Mater..

[8] Wang C., Sim K., Chen J., Kim H., Rao Z., Li Y., Chen W., Song J., Verduzco R., Yu C. (2018). Soft Ultrathin Electronics Innervated Adaptive Fully Soft Robots. Adv. Mater..

[9] Shao J., Xuan M., Zhang H., Lin X., Wu Z., He Q. (2017). Chemotaxis-Guided Hybrid Neutrophil Micromotors for Targeted Drug Transport. Angew. Chem. Int. Ed. Engl..

[10] de Avila B.E., Angsantikul P., Li J., Angel Lopez-Ramirez M., Ramirez-Herrera D.E., Thamphiwatana S., Chen C., Delezuk J., Samakapiruk R., Ramez V. (2017). Micromotor-enabled active drug delivery for in vivo treatment of stomach infection. Nat. Commun..

[11] Beregoi M., Evanghelidis A., Diculescu V.C., Iovu H., Enculescu I. (2017). Polypyrrole Actuator Based on Electrospun Microribbons. ACS Appl. Mater. Interfaces.

[12] Zhang L., Naumov P. (2015). Light- and Humidity-Induced Motion of an Acidochromic Film. Angew. Chem. Int. Ed. Engl..

[13] Lowenberg C., Balk M., Wischke C., Behl M., Lendlein A. (2017). Shape-Memory Hydrogels: Evolution of Structural Principles To Enable Shape Switching of Hydrophilic Polymer Networks. Acc. Chem. Res..

[14] Li J., Thamphiwatana S., Liu W., Esteban-Fernandez de Avila B., Angsantikul P., Sandraz E., Wang J., Xu T., Soto F., Ramez V. (2016). Enteric Micromotor Can Selectively Position and Spontaneously Propel in the Gastrointestinal Tract. ACS Nano.

[15] Wallin T.J., Pikul J., Shepherd R.F. (2018). 3D printing of soft robotic systems. Nat. Rev. Mater..

[16] Sadeghi A., Mondini A., Mazzolai B. (2017). Toward Self-Growing Soft Robots Inspired by Plant Roots and Based on Additive Manufacturing Technologies. Soft Robot..

[17] Truby R.L., Wehner M., Grosskopf A.K., Vogt D.M., Uzel S.G.M., Wood R.J., Lewis J.A. (2018). Soft Somatosensitive Actuators via Embedded 3D Printing. Adv. Mater..

[18] Wehner M., Truby R.L., Fitzgerald D.J., Mosadegh B., Whitesides G.M., Lewis J.A., Wood R.J. (2016). An integrated design and fabrication strategy for entirely soft, autonomous robots. Nature.

[19] Cecchini L., Mariani S., Ronzan M., Mondini A., Pugno N.M., Mazzolai B. (2023). 4D Printing of Humidity-Driven Seed Inspired Soft Robots. Adv. Sci..

[20] Cao X., Xuan S., Sun S., Xu Z., Li J., Gong X. (2021). 3D Printing Magnetic Actuators for Biomimetic Applications. ACS Appl. Mater. Interfaces.

[21] Shin S.R., Migliori B., Miccoli B., Li Y.C., Mostafalu P., Seo J., Mandla S., Enrico A., Antona S., Sabarish R. (2018). Electrically Driven Microengineered Bioinspired Soft Robots. Adv. Mater..

[22] Son H., Park Y., Na Y., Yoon C. (2022). 4D Multiscale Origami Soft Robots: A Review. Polymers.

[23] Jin D.D., Chen Q.Y., Huang T.Y., Huang J.Y., Zhang L., Duan H.L. (2020). Four-dimensional direct laser writing of reconfigurable compound micromachines. Mater. Today.

[24] Kang Y.W., Woo J., Lee H.R., Sun J.Y. (2019). A mechanically enhanced electroactive hydrogel for 3D printing using a multileg long chain crosslinker. Smart Mater. Struct.

[25] Zhang Y.X., Wang Q.Y., Yi S.Z., Lin Z., Wang C.Y., Chen Z.P., Jiang L.L. (2021). 4D Printing of Magnetoactive Soft Materials for On-Demand Magnetic Actuation Transformation. ACS Appl. Mater. Inter..

[26] Cernencu A.I., Lungu A., Dragusin D.M., Stancu I.C., Dinescu S., Balahura L.R., Mereuta P., Costache M., Iovu H. (2021). 3D Bioprinting of Biosynthetic Nanocellulose-Filled GelMA Inks Highly Reliable for Soft Tissue-Oriented Constructs. Materials.

[27] Im G.B., Lin R.Z. (2022). Bioengineering for vascularization: Trends and directions of photocrosslinkable gelatin methacrylate hydrogels. Front. Bioeng Biotechnol..

[28] Wang J.H., Tsai C.W., Tsai N.Y., Chiang C.Y., Lin R.S., Pereira R.F., Li Y.E. (2021). An injectable, dual crosslinkable hybrid pectin methacrylate (PECMA)/gelatin methacryloyl (GelMA) hydrogel for skin hemostasis applications. Int. J. Biol. Macromol..

[29] Li Y.E., Jodat Y.A., Samanipour R., Zorzi G., Zhu K., Hirano M., Chang K., Arnaout A., Hassan S., Matharu N. (2020). Toward a neurospheroid niche model: Optimizing embedded 3D bioprinting for fabrication of neurospheroid brain-like co-culture constructs. Biofabrication.

[30] Cheng K.W., Hsu S.H. (2017). A facile method to prepare superparamagnetic iron oxide and hydrophobic drug-encapsulated biodegradable polyurethane nanoparticles. Int. J. Nanomed..

[31] Ouyang L., Yao R., Zhao Y., Sun W. (2016). Effect of bioink properties on printability and cell viability for 3D bioplotting of embryonic stem cells. Biofabrication.

[32] Wang X., Qin X.H., Hu C., Terzopoulou A., Chen X.Z., Huang T.Y., Maniura-Weber K., Pané S., Nelson B.J. (2018). 3D Printed Enzymatically Biodegradable Soft Helical Microswimmers. Adv. Funct. Mater..

[33] Dong M., Wang X., Chen X.Z., Mushtaq F., Deng S., Zhu C., Torlakcik H., Terzopoulou A., Qin X.H., Xiao X. (2020). 3D-Printed Soft Magnetoelectric Microswimmers for Delivery and Differentiation of Neuron-Like Cells. Adv. Funct. Mater..

[34] Hagihara T., Toyota M. (2020). Mechanical Signaling in the Sensitive Plant Mimosa pudica L. Plants.

[35] Tamiya T., Miyazaki T., Ishikawa H., Iriguchi N., Maki T., Matsumoto J.J., Tsuchiya T. (1988). Movement of water in conjunction with plant movement visualized by NMR imaging. J. Biochem..

[36] Liu H., Dong H., Zhou N., Dong S., Chen L., Zhu Y., Hu H.M., Mou Y. (2018). SPIO Enhance the Cross-Presentation and Migration of DCs and Anionic SPIO Influence the Nanoadjuvant Effects Related to Interleukin-1beta. Nanoscale Res. Lett..

[37] Lakshminarayanan S., Shereen M.F., Niraimathi K.L., Brindha P., Arumugam A. (2021). One-pot green synthesis of iron oxide nanoparticles from Bauhinia tomentosa: Characterization and application towards synthesis of 1, 3 diolein. Sci. Rep..

[38] Jalilian A.R., Panahifar A., Mahmoudi M., Akhlaghi M., Simchi A. (2009). Preparation and biological evaluation of [67 Ga]-labeled-superparamagnetic nanoparticles in normal rats. Rca-Radiochim. Acta.

[39] Kot B.C., Zhang Z.J., Lee A.W., Leung V.Y., Fu S.N. (2012). Elastic modulus of muscle and tendon with shear wave ultrasound elastography: Variations with different technical settings. PLoS ONE.

[40] Wydra R.J., Oliver C.E., Anderson K.W., Dziubla T.D., Hilt J.Z. (2015). Accelerated generation of free radicals by iron oxide nanoparticles in the presence of an alternating magnetic field. Rsc. Adv..

[41] Patra J.K., Baek K.H. (2017). Green biosynthesis of magnetic iron oxide (Fe_3_O_4_) nanoparticles using the aqueous extracts of food processing wastes under photo-catalyzed condition and investigation of their antimicrobial and antioxidant activity. J. Photochem. Photobiol. B.

[42] Rahman O.U., Mohapatra S.C., Ahmad S. (2012). Fe_3_O_4_ inverse spinal super paramagnetic nanoparticles. Mater. Chem. Phys..

[43] Tognato R., Armiento A.R., Bonfrate V., Levato R., Malda J., Alini M., Eglin D., Giancane G., Serra T. (2018). Stimuli-Responsive Nanocomposite for 3D Anisotropic Cell-Guidance and Magnetic Soft Robotics. Adcanced Funct. Mater..

[44] Jaiswal M.K., Xavier J.R., Carrow J.K., Desai P., Alge D., Gaharwar A.K. (2016). Mechanically Stiff Nanocomposite Hydrogels at Ultralow Nanoparticle Content. ACS Nano.

[45] Tsai P.-J., Lee I.-C., Yen M.-H., Li Y.-C.E. (2021). Development and customization of a concentration gradient microgenerator by extrusion 3D printing for drug testing in laboratory studies. Bioprinting.

[46] (2021). Biological Evaluation of Medical Devices. Part 12: Sample Preparation and Reference Materials.

[47] Chang W.C., Tai A.Z., Tsai N.Y., Li Y.E. (2021). An Injectable Hybrid Gelatin Methacryloyl (GelMA)/Phenyl Isothiocyanate-Modified Gelatin (Gel-Phe) Bioadhesive for Oral/Dental Hemostasis Applications. Polymers.

[48] Mukhtarkhanov M., Perveen A., Talamona D. (2020). Application of Stereolithography Based 3D Printing Technology in Investment Casting. Micromachines.

[49] Roco M., Hersam M., CA M. (2011). Innovative and responsible governance of nanotechnology for societal developmentI. Nanotechnology Research Directions for Societal Needs in 2020.

[50] Ali A., Zafar H., Zia M., Ul Haq I., Phull A.R., Ali J.S., Hussain A. (2016). Synthesis, characterization, applications, and challenges of iron oxide nanoparticles. Nanotechnol. Sci. Appl..

[51] Buzea C., Pacheco I.I., Robbie K. (2007). Nanomaterials and nanoparticles: Sources and toxicity. Biointerphases.

[52] Liu X.Y., Yuk H., Lin S.T., Parada G.A., Tang T.C., Tham E., de la Fuente-Nunez C., Lu T.K., Zhao X.H. (2018). 3D Printing of Living Responsive Materials and Devices. Adv. Mater..

[53] Zheng W.J., An N., Yang J.H., Zhou J.X., Chen Y.M. (2015). Tough Al-alginate/Poly(*N*-isopropylacrylamide) Hydrogel with Tunable LCST for Soft Robotics. ACS Appl. Mater. Int..

[54] Bassik N., Abebe B.T., Laflin K.E., Gracias D.H. (2010). Photolithographically patterned smart hydrogel based bilayer actuators. Polymer.

[55] Kim Y.W., Kim J.E., Jung Y., Sun J.Y. (2019). Non-swellable, cytocompatible pHEMA-alginate hydrogels with high stiffness and toughness. Mat. Sci. Eng. C-Mater..

[56] Lee Y., Song W.J., Sun J.Y. (2020). Hydrogel soft robotics. Mater. Today Phys..

