# Supplementary material for: A Printable Magnetic-Responsive Iron Oxide Nanoparticle (ION)-Gelatin Methacryloyl (GelMA) Ink for Soft Bioactuator/Robot Applications
Source: Polymers (Basel). 2023 Dec 20;16(1):25. doi: 10.3390/polym16010025 (PMC10780401; doi:10.3390/polym16010025)
Supplement: Supplementary file 1 [file polymers-16-00025-s001.zip › polymers-2732931-supplementary.pdf]

Supporting Materials

A Printable Magnetic-Responsive Iron Oxide Nanoparticle (ION)-Gelatin Methacryloyl (GelMA) Ink for Soft Bioactuator/Robot Applications

Han-Wen Yang [1,†], Nien-Tzu Yeh [1,†], Tzu-Ching Chen [1], Yu-Chun Yeh [2], I-Chi Lee [2,*] and Yi-Chen Ethan Li [1,*]

Cell culture

L929 and C2C12 cells were cultured with Dulbecco's modified Eagle's medium supplemented (DMEM) with 10 % FBS, 1 % penicillin, and 1 % streptomycin in a cell incubator. Once the cell density achieved 80 % confluency, the cells were suspended by using a 0.5 % Trypsin solution for 5 mins. The suspended cells were collected by a centrifugal procedure and re-seeded to a new culture flask for passage and further tests.

Cell viability testing

The ION-GelMA hydrogels (0.2 g/mL) were immersed in the culture medium at 37 °C for 24 h. Afterward, the extraction medium of ION-GelMA was collected and then used to culture L929 cells following the ISO10993 protocol. Then, MTT assay (Sigma-Aldrich, St. Louis, MO, USA) was used to evaluate the cellular metabolic activity of cells. The MTT solution was used to coculture with cells for 4 h at 37 °C. Subsequently, the supernatant was removed, and DMSO was used to dissolve the purple crystal in the culture well. After dissolving, the supernatant was collected and measured the absorbance at 570 nm using a microplate reader (Epoch, BioTek, Winooski, VT, USA). In the MTT assay, the samples were collected from three batch-independent experiments for each condition (n = 3/condition), and each condition had six samples for each batch-independent experiment.

Cell morphology identification

Cell morphology was identified by immunofluorescent staining. The cells were fixed in 4 % paraformaldehyde solution at 4 °C for 30 mins. A PBS solution was used to wash the fixed cells 3 times (5 minutes per wash). Then 0.1 % Triton-X 100 was used to treat the fixed cells for 10 mins and washed with PBS 3 times. Then, an antibody, fluorophore-labeled anti-F-actin antibody (Alexa Fluor 488 actin conjugate, Invitrogen, MA, USA) was used to further incubate with C2C12 cells for 2 hours at room temperature. An inverted fluorescence microscope (Leica DMIL LED, Germany) was used for imaging the stained.

Particle size distribution (intensity)

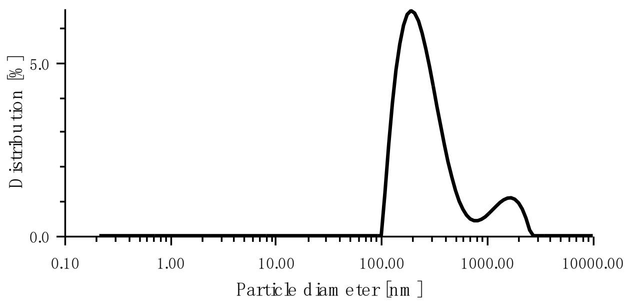

Results

**Hydrodynamic diameter**	329.2 nm	**Mean intensity**	148.7 kcounts/s
**Polydispersity index**	22.7 %	**Absolute intensity**	1033.7 kcounts/s
**Diffusion coefficient**	1.5 µm²/s	**Intercept g1²**	0.8592
**Transmittance**	85.5 %	**Baseline**	1.042

Figure S1. The hydrodynamic diameter of IONs was measured by using DLS size measurement.

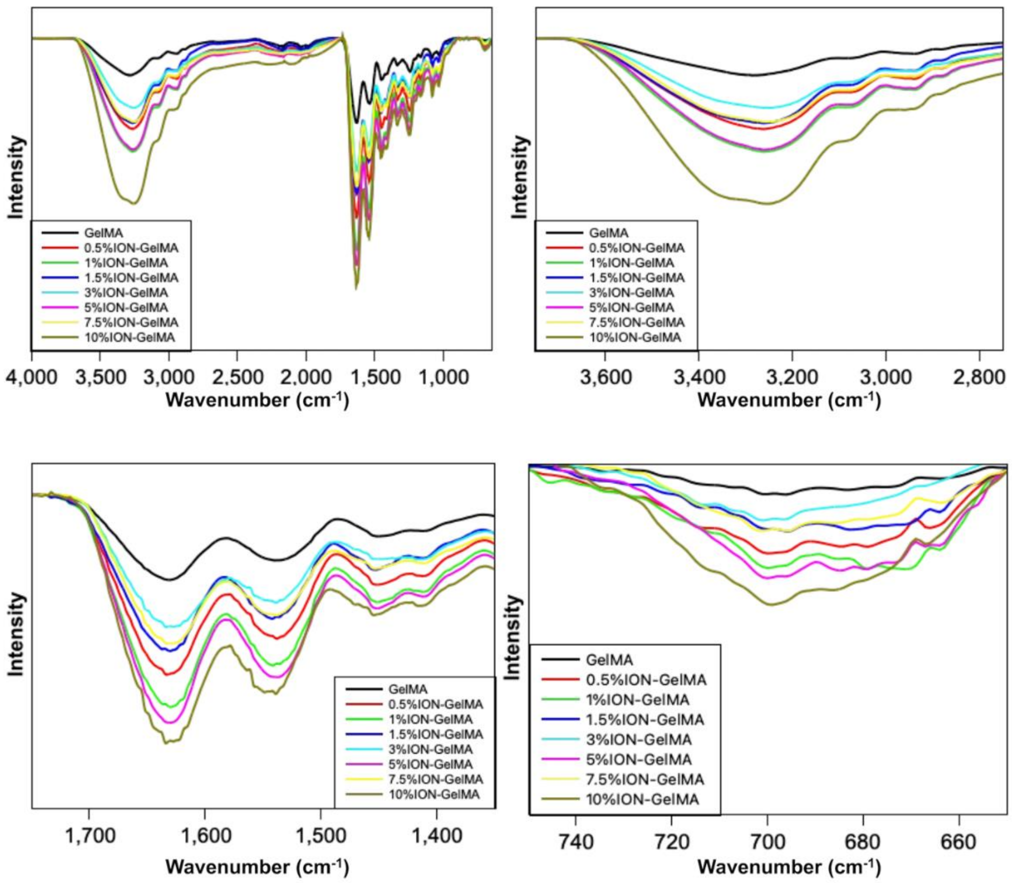

Figure S2. The FTIR spectrum of ION-GelMA inks with different ION concentrations.

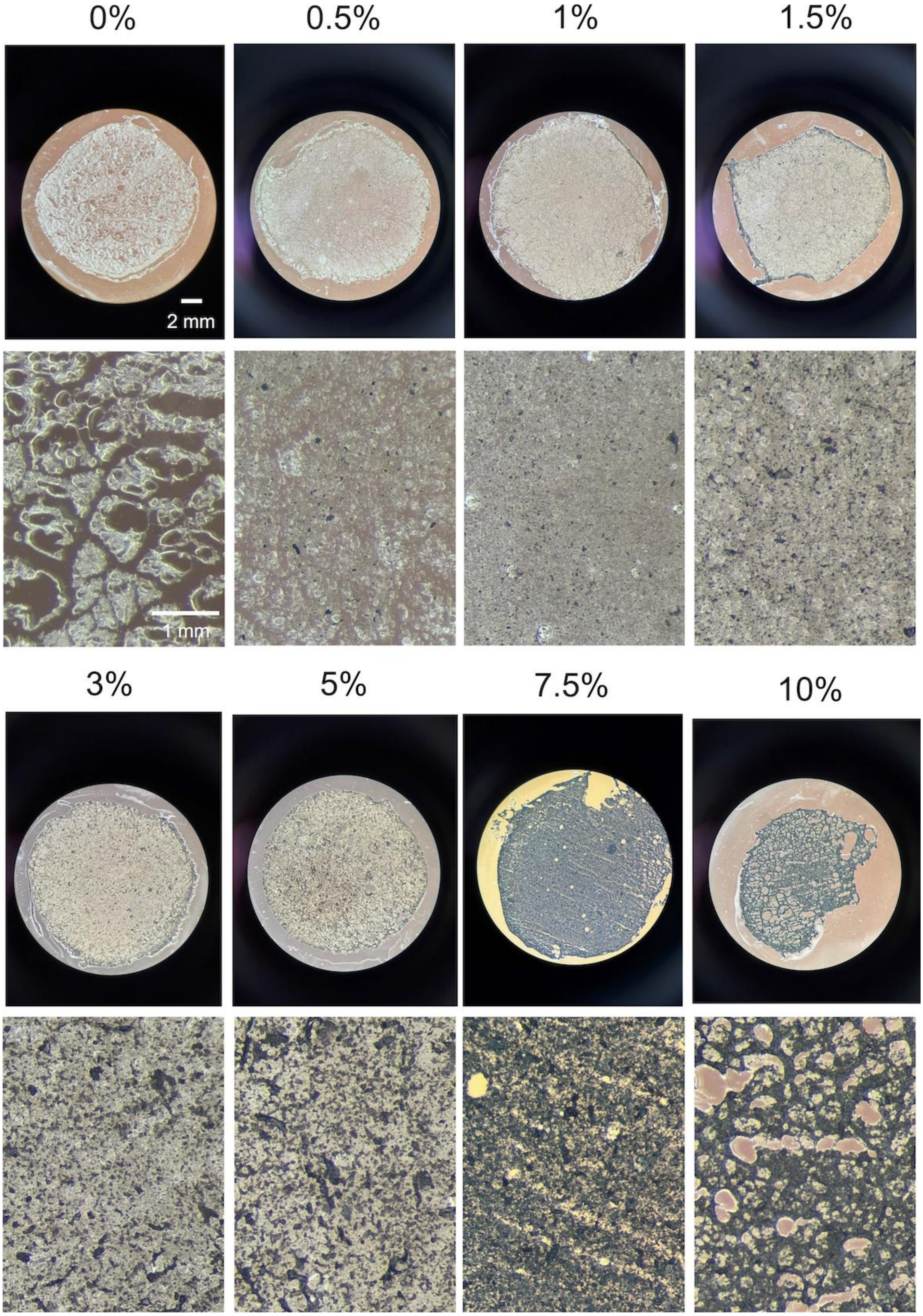

Figure S3. The optical images of ION distribution in the cryosection of GelMA and ION-GelMA hydrogels.

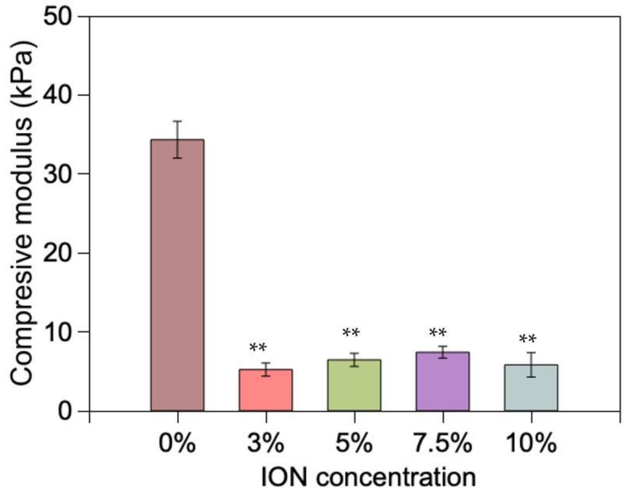

Figure S4. The compressive modulus of ION-GelMA hydrogels with different ION concentrations. (*: comparison with 0% group, **: $p < 0.05$).

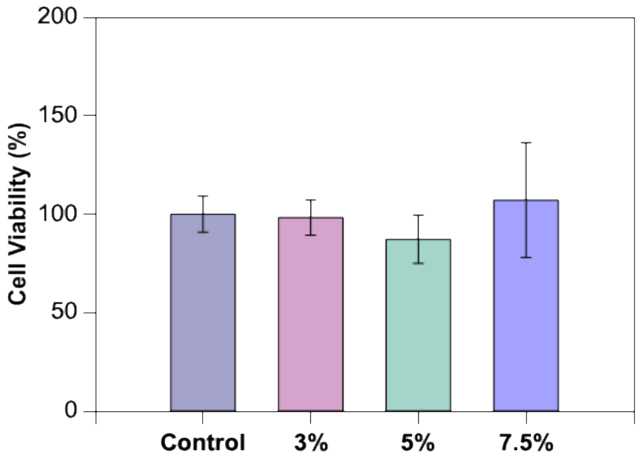

Figure S5. The cell viability of ION-GelMA hydrogels.

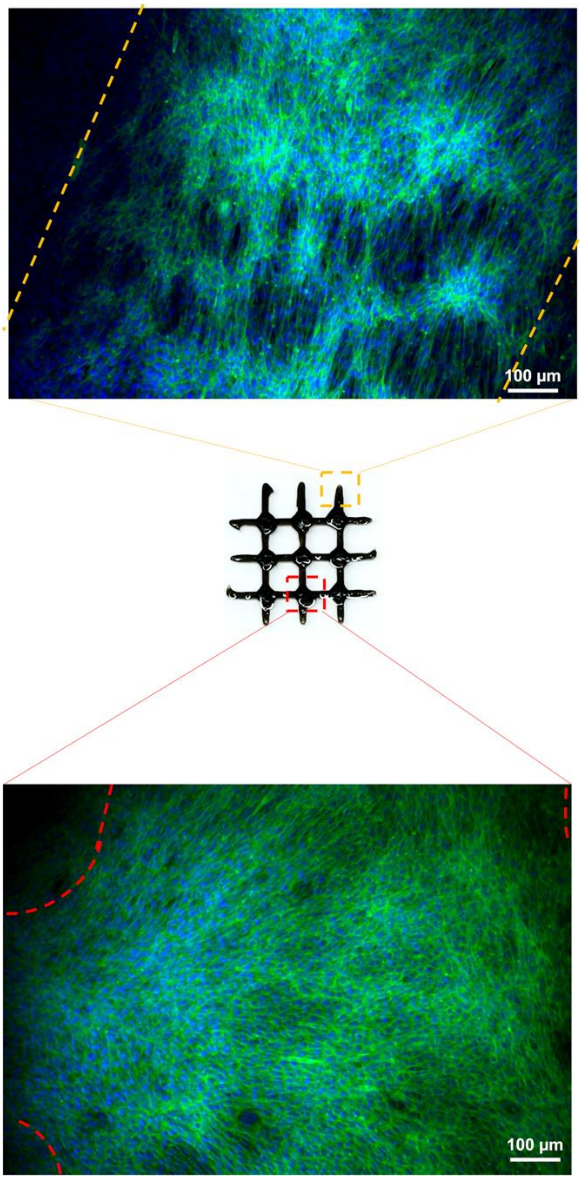

Figure S6. The cell morphology of C2C12 cells were cultured on the surface of printed 5% ION-GelMA structure after 7 days of incubation.
